# Suitable split nitrogen application increases grain yield and photosynthetic capacity in drip-irrigated winter wheat (*Triticum aestivum* L.) under different water regimes in the North China Plain

**DOI:** 10.3389/fpls.2022.1105006

**Published:** 2023-01-13

**Authors:** Abdoul Kader Mounkaila Hamani, Sunusi Amin Abubakar, Zhuanyun Si, Rakhwe Kama, Yang Gao, Aiwang Duan

**Affiliations:** ^1^ Key Laboratory of Crop Water Use and Regulation, Ministry of Agriculture and Rural Affairs/Institute of Farmland Irrigation, Chinese Academy of Agricultural Sciences, Xinxiang, Henan, China; ^2^ Department of Agricultural and Bioresource Engineering, Abubakar Tafawa Balewa University, Bauchi, Nigeria

**Keywords:** drip-fertigation, split N application, wheat yield, photosynthetic capacity, chlorophyll fluorescence

## Abstract

Chemical fertilizer overuse is a major environmental threat, critically polluting soil and water resources. An optimization of nitrogen (N) fertilizer application in winter wheat (*Triticum aestivum* L.) in association with various irrigation scheduling is a potential approach in this regard. A 2-year field experiment was carried out to assess the growth, yield and photosynthetic capacity of drip-irrigated winter wheat subjected to various split applications of urea (240 kg ha^−1^, 46% N). The eight treatments were, two irrigation scheduling and six N application modes in which, one slow-release fertilizer (SRF). Irrigation scheduling was based on the difference between actual crop evapotranspiration and precipitation (ETa-P). The two irrigation scheduling were I_45_ (Irrigation scheduling when ETa-P reaches 45 mm) and I_30_ (Irrigation scheduling when ETa-P reaches 30 mm). The six N levels were N_0-100_ (100% from jointing to booting), N_25-75_ (25% during sowing and 75% from jointing to booting), N_50-50_ (50% during sowing and 50% from jointing to booting), N_75-25_ (75% during sowing and 25% from jointing to booting), N_100-0_ (100% during sowing), and SRF_100_ (240_ kg_ ha^−1^, 43% N during sowing). N top-dressing application significantly (*P<*0.05) influenced wheat growth, aboveground biomass (ABM), grain yield (GY) and its components, photosynthetic and chlorophyll parameters, and plant nutrient content. According to the averages of the two winter wheat-growing seasons, the I_45_N_50-50_ and I_45_SRF_100_ treatments, respectively had the highest GY (9.83 and 9.5 t ha^−1^), ABM (19.91 and 19.79 t ha^−1^), net photosynthetic rate (35.92 and 34.59 µmol m^−2^s^−1^), stomatal conductance (1.387 and 1.223 mol m^−2^s^−1^), *SPAD* (69.33 and 64.03), and chlorophyll fluorescence *F_V_/F_M_* (8.901 and 8.922). The present study provided convincing confirmation that N applied equally in splits at basal-top-dressing rates could be a desirable N application mode under drip irrigation system and could economically compete with the costly SRF for winter wheat fertilization. The I_45_N_50-50_ treatment offers to farmers an option to sustain wheat production in the NCP.

## Introduction

The North China Plain (NCP) is among China’s most essential winter wheat-producing areas and winter wheat (*Triticum aestivum* L.) is one of china’s most important grain crops (Gui et al., 2021). As stated in 2018 by the National Bureau of Statistics of China, the NCP represents ~ 25% of the country’s overall agricultural land, ~ 55% of the country’s overall wheat production area, contributing about 71% of total wheat production, and plays a key role in China’s wheat production (Ye et al., 2022). However, the NCP receives an annual rainfall of 500-700 mm, especially in the summer, and little rainfall during the wheat-growing season, which is insufficient for winter wheat growth and development (Qu et al., 2019). Thus, supplemental irrigation is necessary in the region for the dry-winter and spring seasons to increase wheat yields, which deplete groundwater and negatively affect the environment (Li et al., 2008). In the NCP, an irrigation system with high-performance, for example drip irrigation systems, are generally recommended to overtake environmental problems because they are more performant than outdated irrigation systems (Jha et al., 2017; Zain et al., 2021).

Drip irrigation, as an example of important water-saving irrigation techniques, is beneficial in water-fertilizer resource management (Jha et al., 2019; Yan et al., 2019). Furthermore, drip irrigation enhances crop yield and reduces crop water requirement, tillage costs as well as fertilization doses. Sun et al. (2022) reported that drip irrigation provides crop rhizosphere with sufficient moisture leading to an increase in crop yield. Several studies demonstrated that drip irrigation has a great potentiality for a sustainable development of agricultural in NCP (Si et al., 2020; Zain et al., 2021). To date, drip irrigation has been efficient for large-scale and low-densitycrops, including cotton (Tayel and Mansour, 2013), cash crops (Kirda et al., 2007), corn (Lamm and Trooien, 2003), and fruit production (El-Sayed and El-Hagarey, 2014). However, drip irrigation system with a great water-fertilizer use efficiency is rarely applied to small-scale and high-density crops such as wheat (Bozkurt et al., 2006). Recent studies indicate that irrigation and nitrogen (N) management are required to promote the NCP’s wheat production (Zain et al., 2021; Sun et al., 2022).

The application of fertilizer is another essential input that significantly improves yield and nutritional quality. However, excessive N application results in more than 50% N loss to the environment, which subsequently leads to environmental pollution (Ashraf et al., 2019; Adeel et al., 2021). As a necessary macronutrient, N is needed frequently and in greater quantities than any nutrient (Pan et al., 2019). When N fertilizer is used strategically during wheat production, it could extend the grain-filling stage and increase photosynthetic capacity, thus increasing grain yield (Zhang et al., 2020). However, it was demonstrated that excessive N application induces an increase in N loss and a reduction in grain yield (Tian et al., 2018; Zhao et al., 2019). Previous studies observed that top-dressing N applications can increase grain yield compared to basal application and have revealed that basal N fertilizer application resulted in substantial N loss *via* volatilization (Blandino et al., 2015). Further research has revealed that the highest grain yield obtained the application of a 4:4:2 ratio during the sowing, jointing, and anthesis stages were 11.01% and 9.60% greater than those with a ratio of 6:4 and 4:6 applied during the sowing and the joining stage under conditions where the overall N amount applied was 202.5 kg ha^−1^ (Chen et al., 2018b). Furthermore, slow-release fertilizers (SRFs) are also a category of fertilizer, which contain nutrients (especially N) that are dissolved in water slowly or released slowly (Al-Rawajfeh et al., 2021).

Slow-release fertilizers are a type of fertilizer, which are recognized globally (Al-Rawajfeh et al., 2021). SRFs have low nutrient diversion loss and gradual nutrient release, which is beneficial for crop nutrient uptake and utilization (Shan et al., 2022). Slow-release N fertilizer can meet crops’ overall nutritional requirements of during their growth, decrease the volatilization of ammonia in the field, increase N application efficiency, and decrease environmental contamination (Shan et al., 2022). Crop yields, growth, and development, as well as product quality, can all be improved with SRFs (Chen et al., 2018a; Abubakar et al., 2022). Although, SRFs are costly, many farmers cannot afford them. The COVID outbreak has caused a dramatic increase in fertilizer prices, further driving up the price of agricultural inputs. According to Zhang et al. (2022), there was insufficient evidence to prove that SRFs could completely substitute split N application strategies during wheat production. It is imperative to develop a management strategy that can substitute the conventional fertilization method, either by switching urea for SRFs or by implementing drip-fertigation methods. However, food security may be in peril as the output of food production declines.

Nitrogen is an essential factor for achieving high crop yields due to its impact on the leaf’s photosynthetic capacity (Olszewski et al., 2014). N deficit reduces the capacity of photosystems II and I to transport electrons, which eventually reduces the conversion of photochemical energy. Chlorophyll and photosynthetic capacity are both affected (Živčák et al., 2015). N fertilization directly influences growth, and net photosynthetic rate, and eventually affects yield (Zhang et al., 2021). Optimized N fertilizer application is helpful to enhance wheat leaves’ ability in increasing the *PSII* open part’s ratio and subsequently boost the net photosynthetic rate (Zhang et al., 2021). Limited studies are available on the influences of N fertilizers on wheat’s photosynthetic mechanisms. Under conditions of sufficient soil moisture, the 195 kg N ha^−1^ treatment photosynthetic capacity was greatly increased compared to that of 0 kg N ha^−1^, leading to an increased grain yield (Zheng et al., 2021). Yang et al. (2022) reported that it is also crucial to enhance leaf photosynthesis coupled with N use efficiency (NUE), including N utilisation efficiency and N uptake efficiency. Thus, it is important to investigate the response of photosynthetic characteristics to N fertilizer application under supplementary irrigation conditions.

For optimal agricultural productivity, a proper irrigation schedule and N application modes are crucial. Even though significant work has been done on irrigation scheduling and N application modes during wheat production (Si et al., 2020; Zain et al., 2021). However, deep knowledge of split N application modes under various drip irrigation scheduling in winter wheat is still lacking. Thus, the objectives of this study were (1) to evaluate the changes in leaf gas exchange and chlorophyll fluorescence of winter wheat after anthesis in response to various drip irrigation scheduling and N application rates; (2) to evaluate the influences of different drip irrigation and N scheduling on winter wheat growth and yield; (3) and to find out the optimal drip irrigation scheduling and top-dressing N ratio for suitable wheat production in the NCP. The hypothesis of the current study is that equal split N application at basal-top-dressing rates can sustain or improve wheat physiological growth, aboveground biomass accumulation and yield under the irrigation regime of 45 mm compared to the irrigation quota of 30 mm. The outcomes of the present study will give insights into how drip-irrigated winter wheat’s performance can be improved by adjusting an integrated irrigation and N fertilizer management.

## Materials and methods

### Experimental site and climatic condition

**Figure 1:**
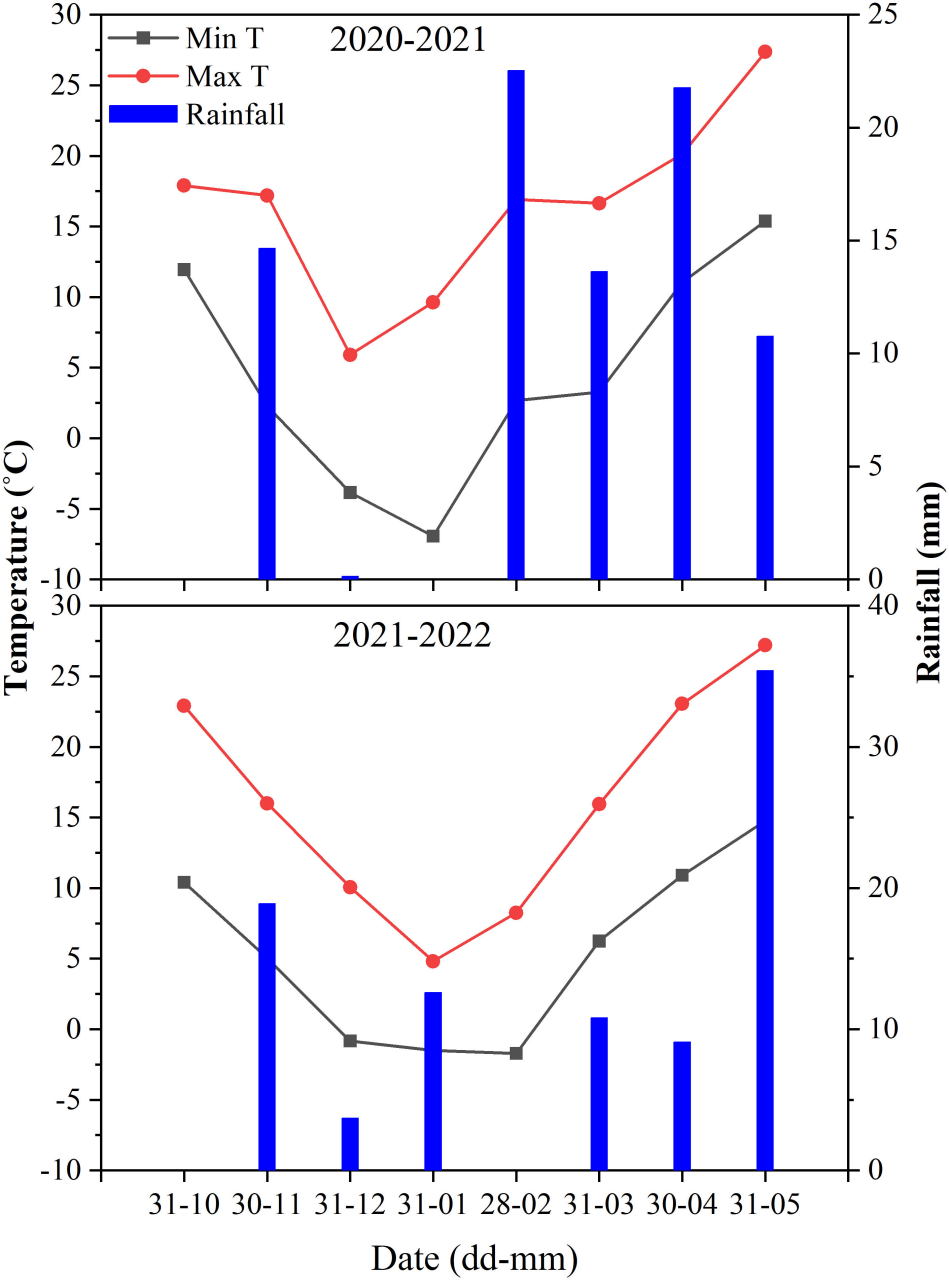
Monthly rainfall and maximum, and minimum temperature during the winter wheat growing season in 2020-2021 and 2021-2022.

The two consecutive winter wheat seasons (2020-2021 and 2021-2022) experiments were conducted at Qiliying Research Site of the Institute of Farmland Irrigation, Chinese Academy of Agricultural Sciences in Xinxiang City, Henan Province, in the NCP (35°08’N, 113° 45’E; altitude 81 m). Warm temperate continental monsoon weather prevails in the area, with an annual average precipitation of 578 mm (~80% of which fall between June and October) and an average precipitation of 161 mm during the wheat-growing season (Si et al., 2020). The overall seasonal precipitation in 2020/2021 and 2021-2022 was 87 and 90.5 mm, respectively. **Figure 1** shows the monthly averages of minimum and maximum temperatures and precipitation for the two wither wheat-growing years. The soil in the research site is a sandy loam. Zain et al. (2021) provided the the experimental area soil’s physical, and chemical characteristics.

### Experimental design and crop management

The winter wheat (*Triticum Aestivum* L.) cultivar sown was a high-yielding variety (Aikang 58) purchased from Danneng Agricultural Materials Company in Xinxiang city; Henan province; P.R China,. The wheat sowing density was 180 kg ha^−1^ (at a normal germination rate with 350-400 plants/m^2^). Two factorial field experiment was conducted adopting a randomized complete block design. The first factor, is the irrigation scheduling with two treatments, while, the second factor is the N application modes with six treatments, including a single treatment of slow-release N-fertilizer (SRF). A total of twelve (2*6) treatments are settled as detailed in **Table 1**. Each treatment was replicated three times. The two irrigation levels were I_45_ (Irrigation scheduling when ETa-P reaches 45 mm) and I_30_ (Irrigation scheduling when ETa-P reaches 30 mm). The six-nitrogen levels were N_0-100_, N_25-75_, N_50-50_, N_75-25_, N_100-0_, and SRF_100_. Duan et al. (2019) and Si et al. (2020) recommended the N (urea) application rate of 240 kg ha^−1^ for winter wheat production. The details of the fertilization schedules are given in **Table 2**. The blocks were divided by 0.5 m access lines, and the plot was 15 m by 3 m. Using a tractor-drawn rotary cultivator, the soil was cultivated to a depth of 20 cm, and then leveled with a harrow. The sowing of winter wheat occurred on October 24, 2020, and October 22, 2021. The harvest dates were June 2, 2020, and June 3, 2021. Urea (46% N), calcium superphosphate (16% P_2_O_5_), and potassium sulfate (50% K_2_O) were used to apply N, P, and K fertilizers, respectively. At sowing, P and K fertilizers were applied at the rate of 120 kg ha^−1^, while N was applied at the sowing, jointing, and booting stages of wheat growth.

**Table 1:** Experimental treatment labels with different irrigation scheduling and nitrogen application modes.

Treatment label	Signification
I_30_	Irrigation scheduling when ETa-P reaches 30 mm
I_45_	Irrigation scheduling when ETa-P reaches 45 mm
N_0-100_	100% of N at jointing/booting
N_25-75_	25% of N at sowing, and 75% of N at jointing/booting
N_50-50_	50% of N at sowing, and 50% of N at jointing/booting
N_75-25_	75% of N at sowing, and 25% of N at jointing/booting
N_100-0_	100% of N at sowing
SRF_100_	100% of slow-release fertilizer (SRF, 43% N, 240 kg ha^−1^) at sowing

**Table 2:** Fertilizer application schedules of the experimental treatments.

Fertilization events	Fertilizer application rate (kg ha^-1^)					
	N_0-100_	N_25-75_	N_50-50_	N_75-25_	N_100-0_	SRF_100_
sowing	0	60	120	180	240	240
jointing stage	120	90	60	30	0	0
booting stage	120	90	60	30	0	0

### Irrigation and fertigation methods

Installation of a surface drip irrigation system with 60 cm lateral irrigation line spacing was done with drippers 20 cm spaced along the laterals. The discharge rate of drippers was 2.2 L h^−1^ at 0.10–0.15 MPa working pressure. Each plot had a flow meter installed to control the amount of irrigation water released. Eq. (1) was used to calculate crop evapotranspiration between two irrigation episodes:


(1) $$ET_{a}=\;K_{c}\times \;ET_{o}$$

where *ET_a_* = Actual crop evapotranspiration (mm d^−1^), *K_c_* = Crop coefficient (according to Gao et al. (2009)), early season, mid-season, and late season *K_c_* are 0.36, 1.19, and 0.28, respectively). The reference evapotranspiration (*ET_o_*) was determined following Allen et al. (1998). The irrigation necessity (I) was computed using Equation (2):


(2) $$I\;=\;ET_{a}–\;Rainfall$$

Irrigation episodes occurred whenever the overall I reached 45 or 30 mm depending on experimental treatments as suggested by Shen et al. (2020).

Using a closed-tank fertigation system, topdressing fertilization occurred during the winter wheat jointing/booting stages (Abubakar et al., 2022). The SRF used in this study was produced through the polymer coating process. The coating material was polyolefin polymer resin with talcum powder as an additive. The SRF coating accounted for 5.6% of the SRF mass, the N concentration was 43%, and the release duration was 30 days (the required number of days for the SRF to release 80% of its N at 25˚C).

### Field sampling and measurements

#### Determination of growth and yield-related parameters

Plant height was measured from the ground surface to plant’s tip. The spikelet was included in the plant height during the later stages of wheat growth. Winter wheat plant height and leaf area index (LAI) were recorded at 10 to 15-day intervals from 10 randomly selected plants in each plot. The method described by Zain et al. (2021) was used to calculate LAI. A ruler was used to measure the leaf’s length and width of each leaf from the 10 randomly selected plants, and the leaf area per plant (LA) was determined using the following equation and presented in m^2^:


(3) $$\text{Leaf area per plant }\left(\text{A}\right)\;=\;\frac{\sum_{\text{i }=\;1}^{\text{n}}{\text{A}}_{\text{i}}}{\text{n}}\;=\;\frac{\sum_{\text{i }=\;1}^{\text{n}}\left[\sum_{\text{j }=\;1}^{\text{m}}\left({\text{ L}}_{\text{i}}\;\times {\text{ W}}_{\text{j}}\right)\;\times \;0.80\right]}{\text{n}}$$

(4) $$\text{LAI }=\;\frac{\text{A }\times \text{ N}}{\text{s}}$$

where n denotes the number of plant samples used to calculate LA (n = 10); A_i_ is the leaf area of the i^th^ plant; m is the number of leaves in the i^th^ plant, and L_j_ and W_j_ are the length and width of the j^th^ leaf in the i^th^ plant (both in cm). N denotes the number of plants (including tillers) in 1 m of the row, and S denotes row spacing (S = 0.2 m). At harvest, 10 plants in each plot were taken to determine plant height, and yield components, including spike length, the number of grains per spike, thousand-grain weight, and the number of grains per 10 plants. Finally, for each experimental plot, a plants’ 1 m^2^ area was sampled to determine the grain yield (t ha^−1^) and aboveground biomass (t ha^−1^). Each experimental plots’ grain yield was determined by weighing grains after naturally dried to 12% moisture content. The harvest index (HI) was calculated using the following equation:


(5) $$HI=\frac{Grain\;yield\;\left(t\;ha^{-1}\right)}{Aboveground\;biomass\;\left(t\;ha^{-1}\right)}$$

#### Determination of gas exchange and chlorophyll parameters

Gas exchange characteristics, such as net photosynthetic rate (*A_n_*) and stomatal conductance (*g_s_*), were recorded using the LI-6400XT portable gas exchange measuring system (LI-COR, Lincoln Nebraska, USA). Three selected leaves in each plot were measured between 9:00 and 11:00 am at 0, 7, 14, 21, and 28 days after anthesis (DAA) under the condition of 25°C, 400 μmol mol^−1^ CO_2_ concentration, 500 μmol s^−1^ flow rate, and 1300 mol m^−2^ s^−1^ leaf chambers’ photosynthetic active radiation (Zheng et al., 2021). The intrinsic water use efficiency (WUEi) was computed as *A_n_*/*g_s_*. A Minolta SPAD-502 Chlorophyll Meter was used to determine the chlorophyll content, which determines SPAD values proportional to the chlorophyll content according to the leaf transmittance (Mehrabi and Sepaskhah, 2022). After dark adaptation for 30 min, the maximum quantum efficiency (*F_V_*/*F_M_*) was determined according to Xu et al. (2017).

#### Determination of plant nutrient content

Plant nutrient content, including total nitrogen (TN), total phosphorus (TP), and total potassium (TK) was determined at the Key Laboratory of Crop Water Use and Regulation, Institute of Farmland Irrigation/Chinese Academy of Agricultural Sciences, Xinxiang, Henan, P.R. China. TN content was determined in plant samples using the Kjeldhal method, as described by Bremner (1996). TP concentration was measured using a standard method (Misra, 1968). Plant TK concentration was extracted using a mixture of HNO_3_^−^H_2_SO_4_^−^HCLO_4_ by digestion and determined using a flame photometer as described by Jackson (1973).

### Statistical analysis

Standard ANOVA was used to perform the statistical analysis in SPSS 22.0. The least significant difference (LSD) test was used to compare the treatments among each other at a significance level of 0.05. Two-way ANOVA was performed where irrigation scheduling and N application modes were used as the main factors. Person’s correlation was used to evaluate the relationship between the wheat grain yield and various parameters. The graphs were constructed using Origin-Pro 2021b (Origin Lab, Northampton, MA, USA).

## Results

### Seasonal variation of crop growth

**Figure 2:**
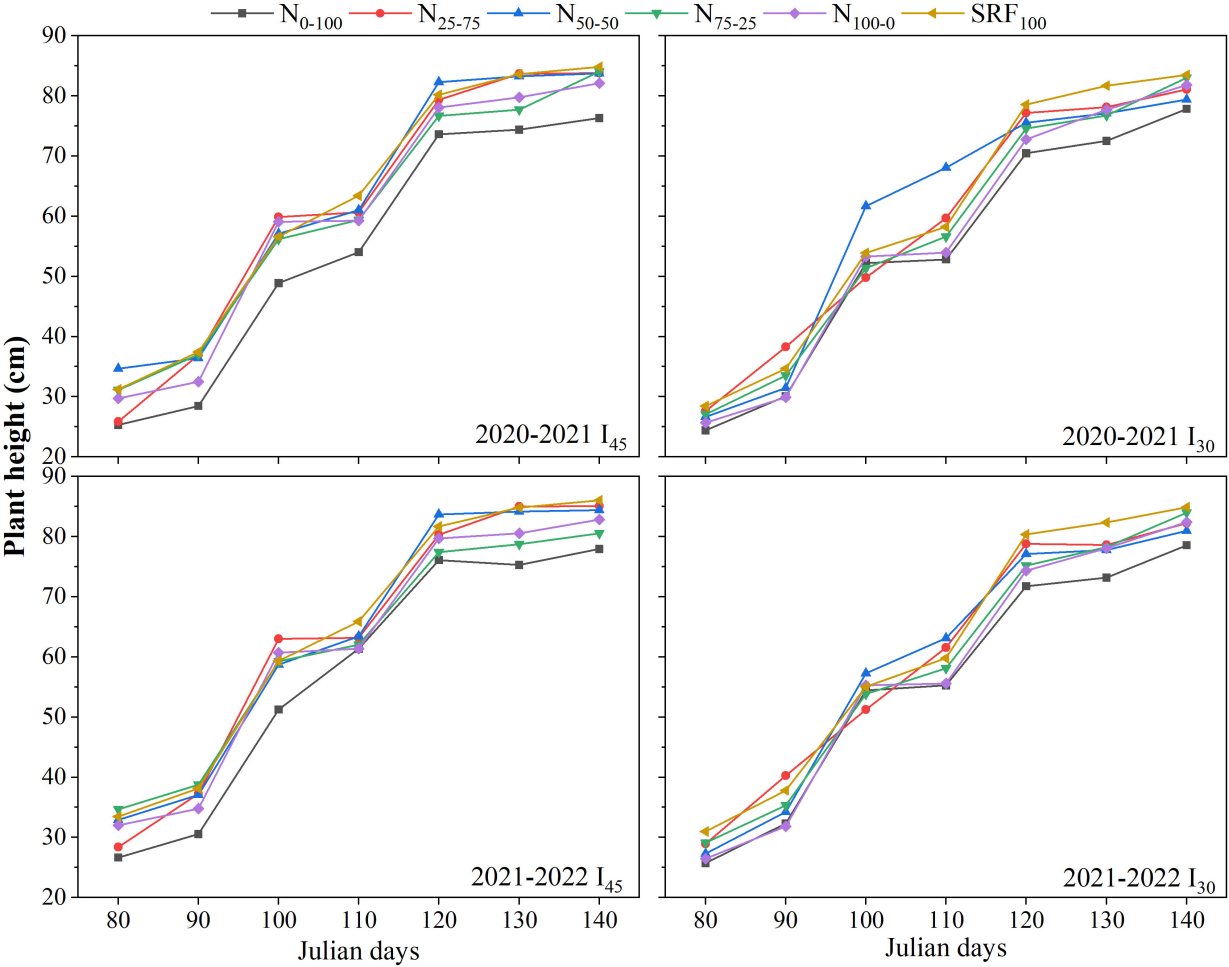
Variation of plant height under different irrigation scheduling and nitrogen application modes during 2020-2021 and 2021-2022 winter wheat-growing seasons.

**Figure 3:**
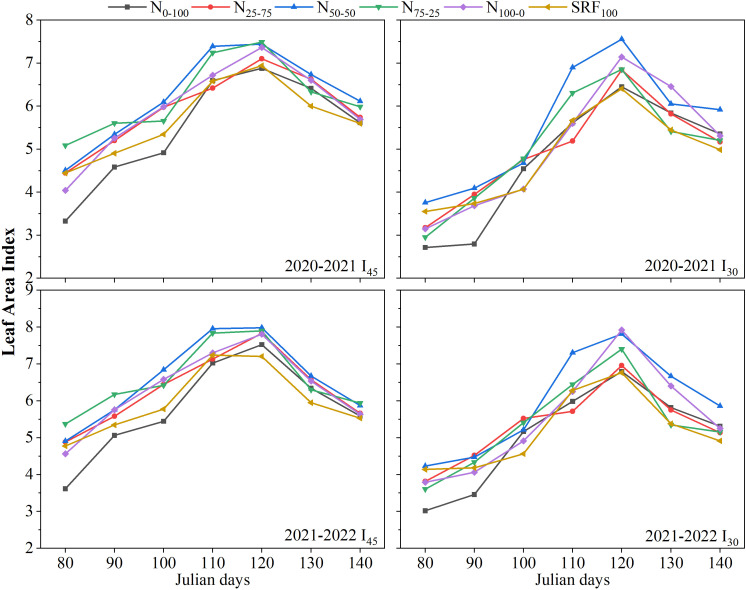
Variation of leaf area index under different irrigation scheduling and nitrogen application modes during 2020-2021 and 2021-2022 winter wheat-growing seasons.

The temporal variations in plant growth parameters (plant height and leaf area index) of winter wheat under various N and irrigation scheduling during the 2020-2021 and 2021-2022 growing seasons are presented in **Figures 2**, **3**. The curves of plant height shows similar trend under the various N and irrigation scheduling during both wheat-growing seasons (**Figure 2**). From all the experimental treatments, plant height increases from sowing to reach its maximum at the maturity of winter wheat. At winter wheat maturity, the highest plant height was observed in SRF_100_ treatment under both irrigation regimes and during both growing seasons. The lowest plant height was obtained with N_0-100_ treatment under both irrigation regime and during both growing seasons (**Figure 2**).

The curves for leaf area index (LAI) showed similar patterns under the various N and irrigation scheduling during both wheat-growing seasons (**Figure 3**). With all the experimental treatments, LAI increases from sowing to reach its maximum at around 120 Julian days of the growing season and then decrease. At winter wheat maturity, the highest LAI was observed in N_50-50_ treatment under both irrigation regimes and during both growing seasons. The lowest LAI index was observed in N_0-100_ treatment at early growth stages (returning green and jointing) under the different irrigation scheduling and during both growing seasons (**Figure 3**). The optimum variations in plant height and LAI were observed from the I_45_N_50-50_ treatment during both winter wheat growing seasons.

### Grain yield and its components

Different N and irrigation scheduling significantly (*P<*0.05) affect grain yield during the two winter wheat growing seasons (**Table 3**). During both winter wheat-growing seasons, the I_45_N_50-50_ treatment showed the highest grain yield with 9.72 and 9.94 t ha^−1^ in 2020-2021 and 2021-2022, respectively. The grain yield of I_45_N_50-50_ and I_45_SRF_100_ were significantly (*P<*0.05) similar during both seasons. The lowest grain yield was obtained with the I_30_N_0-100_ during both growing seasons with 7.08 and 7.53 t ha^−1^ in 2020-2021 and 2021-2022, respectively. Across the two winter wheat-growing seasons, different irrigation scheduling and N application modes significantly affected winter wheat grain yield, but their interaction was insignificant (**Table 3**).

**Table 3:** Influence of different irrigation scheduling and nitrogen application modes on winter wheat grain yield (t ha^−1^) during 2020-2021 and 2021-2022 growing seasons.

Season	Treatment	N_0-100_	N_25-75_	N_50-50_	N_75-25_	N_100-0_	SRF_100_
	I_45_	7.23^de^	9.02^ab^	9.72^a^	8.23^bcd^	8.11^bcde^	9.12^ab^
	I_30_	7.08^e^	8.07^bcde^	8.53^bc^	7.59^cde^	7.51^cde^	8.11^bcde^
2020-2021	I	**					
	N	***					
	I × N	ns					
	I_45_	7.79 ^cd^	9.43^ab^	9.94^a^	8.79^abcd^	8.66^abcd^	9.88^a^
2021-2022	I_30_	7.53^d^	8.54 ^bcd^	9.01^abc^	7.87^cd^	8.09^cd^	8.66^abcd^
	I	**					
	N	**					
	I × N	ns					

I; Irrigation level, N; Nitrogen application mode, Each value indicates the mean of three replicates and the different letters within the row and column represent a significant difference at P< 0.05. Significance level = ns (non-significant at P< 0.05), * (P< 0.05), ** (P< 0.01), and *** (P< 0.001).

Data given in **Table 4** presents the influence of different irrigation scheduling and N application modes on winter wheat yield components during the two consecutive growing seasons. Compared with the N_100-0_ treatment under different irrigation regimes and during both growing seasons, the split applications of N significantly (*P<*0.05) increased yield components, including spike length (SL), number of grains per spike (GS), and thousand-grain weight (TGW). I_45_N_50-50_ treatment significantly (*P<*0.05) increased the number of grains per 10 plants (G 10 plants^−1^) when compared to I_45_N_0-100_, I_30_N_0-100_, and I_30_N_100-0_ during both winter wheat-growing seasons. Across the two winter wheat-growing seasons, except for the number of grains per 10 plants, different irrigation scheduling and N application modes significantly affected winter wheat yield components, but their interaction was insignificant (**Table 4**). The I_45_N_50-50_ treatment results in the highest grain yield and yield components during both winter wheat growing seasons.

**Table 4:** Interactive effect of different irrigation scheduling and nitrogen application modes on winter wheat yield components during 2020-2021 and 2021-2022 growing seasons.

Season	2020-2021				2021-2022			
Treatment	SL (cm)	GS	TGW (g)	G 10plants^−1^	SL (cm)	GS	TGW (g)	G 10plants^−1^
I45N0-100	7.74^de^	31.66^d^	48.29^de^	374.3^b^	7.79^de^	33.05^de^	49.66^ef^	378.8^b^
145N25-75	8.81^ab^	36.60^abc^	51.67^abc^	425^ab^	8.88^a^	37.02^abc^	52.89^abcd^	430.9^ab^
I45N50-50	8.93^a^	38.56^a^	52.95^a^	448.33^a^	8.98^a^	39.35^a^	53.85^a^	451.23^a^
I45N75-25	8.75^ab^	35.93^bc^	51.62^abc^	410^ab^	8.81^ab^	36.60^bc^	53.20^ab^	412.4^ab^
I45N100-0	8.72^ab^	35.67^bc^	51.42^abc^	403^ab^	8.76^ab^	36.56^bc^	52.01^abcde^	406.9^ab^
I45SRF100	8.92^a^	36.96^ab^	52.34^ab^	426^ab^	8.97^a^	37.85^ab^	53.07^abc^	432.7^ab^
I30N0-100	7.28^e^	29.53^d^	47.72^e^	372^b^	7.34^e^	30.99^e^	48.98^f^	377.7^b^
I30N25-75	8.50^abc^	35.20^bc^	49.73^cde^	390.3^ab^	8.56^abc^	36.47^bc^	50.64^bcdef^	396.6^ab^
I30N50-50	8.69^ab^	35.60^bc^	50.89^abc^	402.3^ab^	8.71^ab^	36.39^bc^	52.43^abcd^	407.9^ab^
I30N75-25	8.33^bc^	34.80^bc^	49.66^cde^	389.7^ab^	8.36^bc^	35.74^bc^	50.47^cdef^	393.8^ab^
I30N100-0	8.04^cd^	34.53^c^	49.48^cde^	379.7^b^	8.06^cd^	34.98^cd^	50.38^def^	386.2^b^
I30-SRF100	8.62^ab^	35.60^bc^	50.45^bcd^	394.3^ab^	8.69^ab^	36.63^bc^	51.57^abcdef^	398.6^ab^
I	***	**	**	*	***	**	**	ns
N	***	***	**	ns	***	***	**	ns
I × N	ns	ns	ns	ns	ns	ns	ns	ns

SL; Spike length, GS; Number of grains per spike, TGW; Thousand-grain weight, G 10plants^−1^; Number of grains per ten plants. Each value indicates the mean of three replicates and the different letters within a column represent a significant difference at P< 0.05. Significance level = ns (non-significant at P< 0.05), * (P< 0.05), ** (P< 0.01), and *** (P< 0.001).

### Aboveground biomass and harvest index

The influence of different irrigation scheduling and N application modes on winter wheat aboveground biomass (ABM) during the 2020-2021 and 2021-2022 growing seasons is presented in **Table 5**. The winter wheat ABM accumulation shows a similar trend during both seasons. Compared to N_0-100_, the N fertigation rate of N_50-50_ significantly (*P<*0.05) increased the wheat ABM under both water regimes and growing seasons. The I_45_N_50-50_ treatment shows the highest ABM accumulation with 19.41 and 20.41 t ha^−1^ in 2020-2021 and 2021-2022, respectively. The I_30_N_0-100_ treatment shows the lowest ABM accumulation with 12.45 and 13.44 t ha^−1^ in 2020-2021 and 2021-2022, respectively. Across the two winter wheat-growing years, different irrigation scheduling and N application modes significantly affected winter wheat ABM accumulation, but their interaction was insignificant (**Table 5**).

**Table 5:** Influence of different irrigation scheduling and nitrogen application modes on winter wheat aboveground biomass (t ha^−1^) during 2020-2021 and 2021-2022 growing seasons.

Season	Treatment	N_0-100_	N_25-75_	N_50-50_	N_75-25_	N_100-0_	SRF_100_
2020-2021	I_45_	14.69^ef^	19^ab^	19.41^a^	18.72^abc^	17.73^abcd^	19.11^ab^
	I_30_	12.45^f^	16.53^bcde^	18.46^abcd^	16.32^cde^	15.89^de^	17.19^abcde^
	I	***					
	N	***					
	I × N	ns					
2021-2022	I_45_	15.36^cd^	19.45^ab^	20.41^a^	19.79^ab^	19.39^ab^	20.47^a^
	I_30_	13.44^d^	17.65^abc^	18.95^ab^	17.19^bc^	17.38^bc^	18.41^ab^
	I	**					
	N	***					
	I × N	ns					

I, Irrigation level; N, Nitrogen application mode; Each value indicates the mean of three replicates and the different letters within the row and column represent a significant difference at P< 0.05. Significance level = ns (non-significant at P< 0.05), * (P< 0.05), ** (P< 0.01), and *** (P< 0.001).

Data presented in **Table 6** indicates the Influence of different irrigation scheduling and N application modes on winter wheat harvest index (HI) during the 2020-2021 and 2021-2022 growing seasons. The I_30_N_0-100_ treatments show the highest HI of 0.58 and 0.58 in 2020-2021 and 2021-2022, respectively and then follows by I_45_N_50-50_ with a HI of 0.50 and 0.48 in 2020-2021 and 2021-2022, respectively. Across the two winter wheat-growing years, different irrigation scheduling and N application modes as well as their interactions insignificantly affected winter wheat HI (**Table 6**). During both winter wheat growing seasons, the I_45_N_50-50_ treatment shows the highest ABM accumulation, while the I_30_N_0-100_ treatment shows the highest HI.

**Table 6:** Influence of different irrigation scheduling and nitrogen application modes on winter wheat harvest index during 2020-2021 and 2021-2022 growing seasons.

Season	Treatment	N_0-100_	N_25-75_	N_50-50_	N_75-25_	N_100-0_	SRF_100_
2020-2021	I_45_	0.49^ab^	0.47^b^	0.50^ab^	0.44^b^	0.46^b^	0.47^b^
	I_30_	0.58^a^	0.48^ab^	0.46^b^	0.46^b^	0.47^b^	0.47^b^
	I	ns					
	N	ns					
	I × N	ns					
2021-2022	I_45_	0.50^ab^	0.48^ab^	0.48^ab^	0.44^b^	0.44^b^	0.48^ab^
	I_30_	0.58^a^	0.48^ab^	0.47^ab^	0.45^b^	0.46^ab^	0.47^ab^
	I	ns					
	N	ns					
	I × N	ns					

I, Irrigation level; N, Nitrogen application mode; Each value indicates the mean of three replicates and the different letters within the row and column represent a significant difference at P< 0.05. Significance level = ns stands for non-significant at P< 0.05.

### Leaf photosynthetic parameters

**Figure 4:**
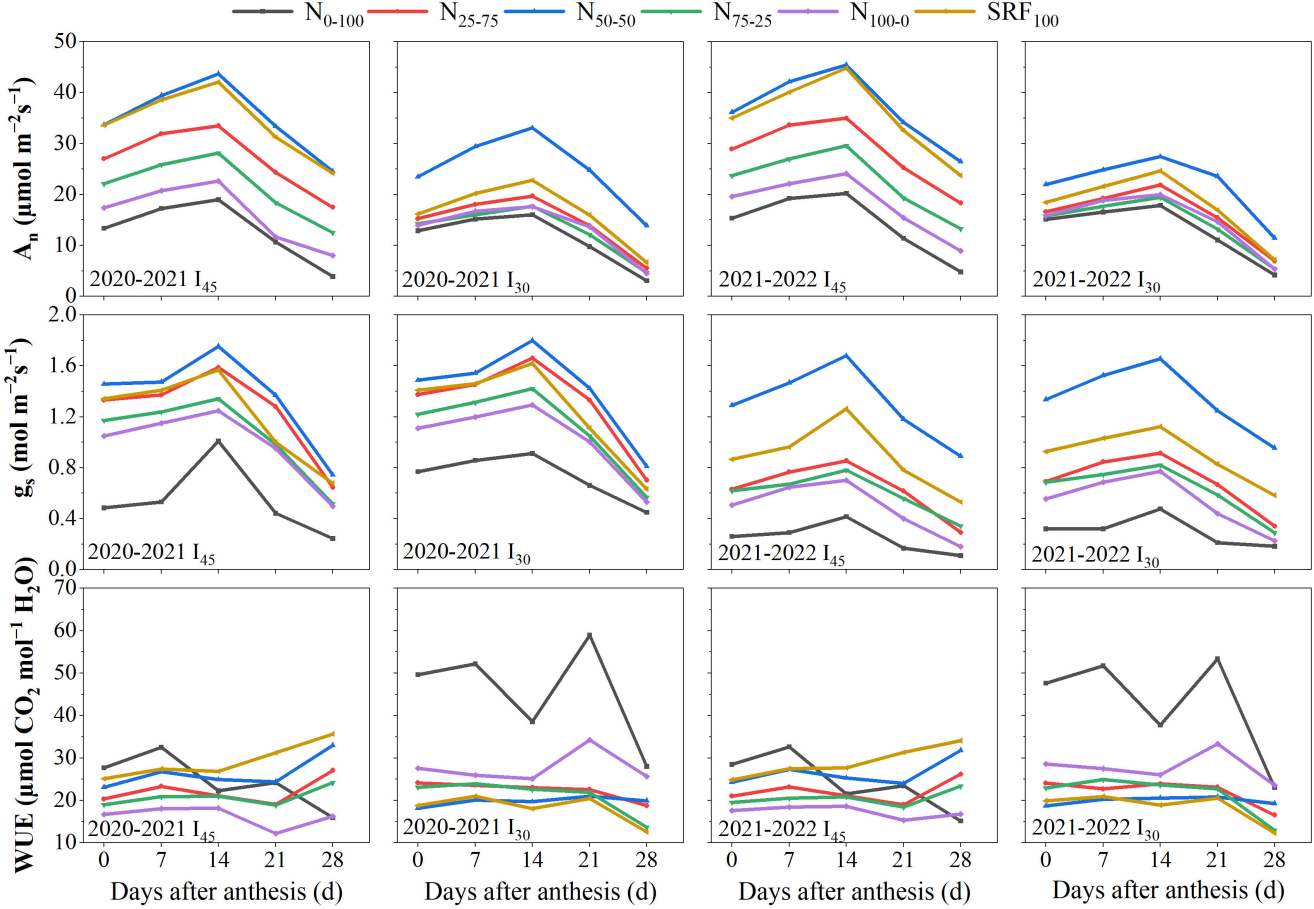
Effects of different irrigation scheduling and nitrogen application modes on photosynthetic capacity during 2020-2021 and 2021-2022 winter wheat-growing seasons. I45 = Irrigation scheduling at 45 mm, I30 = Irrigation scheduling at 30 mm.

Gas exchange charactheristics, including net photosynthetic rate (*A_n_*), stomatal conductance (*g_s_*), and instantaneous water use efficiency (*iWUE*) of winter wheat leaves were affected by different irrigation scheduling and split N applications (**Figure 4**). From 0 to 14-days after anthesis, *A_n_* and *g_s_* continually rise to reach their maximum from all experimental treatments under different irrigation scheduling and N application modes and during both growing seasons. From 14 to 28-days after anthesis, *A_n_* and *g_s_* continually decrease from all experimental treatments under different irrigation scheduling and N application modes and during both growing seasons. From 0 to 28-days after anthesis, the highest values of *A_n_* and *g_s_* were observed in the N_50-50_ and SRF_100_ treatments under different irrigation scheduling and N application modes and during both growing seasons. From 0 to 28-days after anthesis, the N_0-100_ and SRF_100_ treatments show the highest values of *WUEi* from all experimental treatments under 30 mm irrigation scheduling with different N application modes and during both growing seasons (**Figure 4**). The post anthesis gas exchange charactheristics remain higher under the I_45_N_50-50_ treatment during both winter wheat growing seasons.

### SPAD chlorophyll and chlorophyll fluorescence

**Figure 5:**
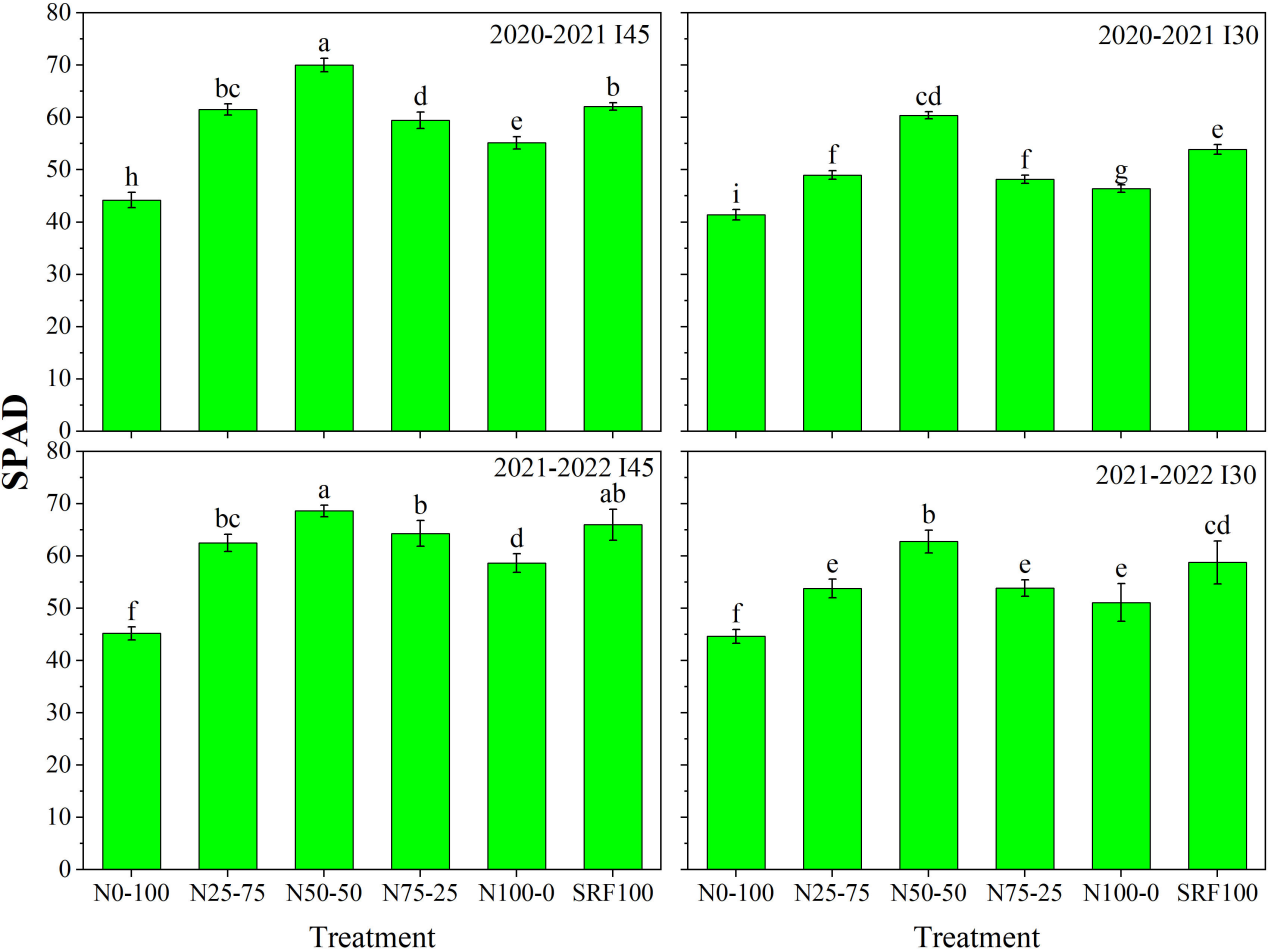
Effects of different irrigation scheduling and nitrogen application modes on SPAD chlorophyll during 2020-2021 and 2021-2022 winter wheat-growing seasons. Each value indicates the mean of three replicates ± standard deviation and the different letters on top of the error bar represent a significant difference at *P*< 0.05.

Data presented in **Figure 5**, indicate that different irrigation scheduling and N application modes significantly (*P<*0.05) affected winter wheat SPAD chlorophyll content. Except for the I_45_SRF_100_ treatment, the I_45_N_50_-_50_ treatment significantly (*P<*0.05) increased the SPAD chlorophyll content compared to other experimental treatments during both growing seasons. The I_45_N_50_-_50_ treatment shows the highest SPAD chlorophyll content with 70.05 and 68.61 in 2020-2021 and 2021-2022, respectively. The I_30_N_0_-_100_ treatment shows the lowest SPAD chlorophyll content with 41.38 and 44.63 in 2020-2021 and 2021-2022, respectively. Winter wheat SPAD chlorophyll content was significantly (*P<*0.05) decreased by 40.92 and 34.95% in 2020-2021 and 2021-2022, respectively when comparing I_45_N_50_-_50_ to I_30_N_0_-_10_ treatment.

**Figure 6:**
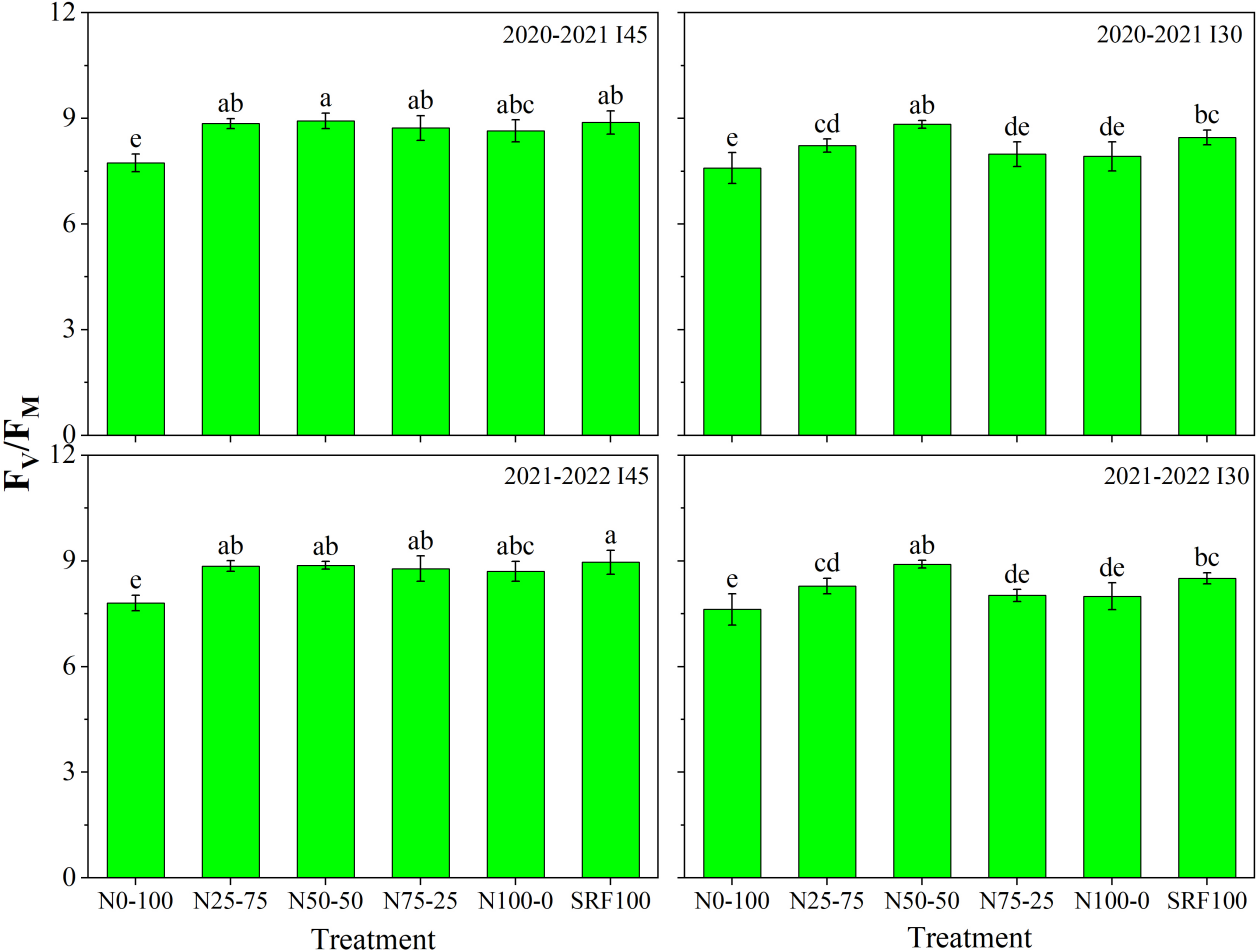
Effects of different irrigation scheduling and nitrogen application modes on the maximal photochemical efficiency of photosystem II (F_V_/F_M_) during 2020-2021 and 2021-2022 winter wheat-growing seasons. Each value indicates the mean of three replicates ± standard deviation and the different letters on top of the error bar represent a significant difference at *P*< 0.05.

Chlorophyll fluorescence (*F_V_/F_M_*) was significantly (*P<*0.05) affected by different irrigation scheduling and N application modes during both growing seasons (**Figure 6**). During the 2020-2021 winter wheat-growing season, compared to the I_45_N_50_-_50_ treatment, *F_V_/F_M_* was significantly (*P<*0.05) decreased by 15.05% under the I_30_N_0_-_100_ treatment. During the 2021-2022 growing season, compared to the I_45_SRF_100_ treatment, *F_V_/F_M_* was significantly (*P<*0.05) decreased by 14.95% in the I_30_N_0_-_100_ treatment. The I_45_N_50_-_50_ treatment shows had the highest value (8.932) of *F_V_/F_M_* in 2020-2021, while the I_45_SRF_100_ treatment shows the highest value (8.961) of *F_V_/F_M_* in 2021-2022. The I_30_N_0_-_100_ treatment shows the lowest values of *F_V_/F_M_* with 7.587 and 7.621 in 2020-2021 and 2021-2022, respectively. (**Figure 6**). The I_45_N_50_-_50_ treatment shows the highest SPAD chlorophyll content and (*F_V_/F_M_*) during both winter wheat growing seasons.

### Winter wheat nutrient content

As shown in **Table 7**, during the 2020-2021 and 2021-2022 winter wheat-growing years, the interactive effect of different irrigation scheduling and N application modes significantly (*P<*0.05) affected winter wheat nutrient content, including total nitrogen (TN), total phosphorus (TP), and total potassium (TK). Compared to the I_30_N_0-100_ treatment, the I_45_N_50-50_ treatment significantly (*P<*0.05) enhanced the TN content by 42.48 and 35.81% in 2020-2021 and 2021-2022, respectively. The I_45_SRF_100_ treatment significantly (*P<*0.05) increased the TP content by 38.4 and 36.12% in 2020-2021 and 2021-2022, respectively in comparison with the I_30_N_0-100_ treatment. With the application of The I_45_N_50_-_50_ treatment, the TK content significantly (*P<*0.05) increased by 31.45 and 32.61% in 2020-2021 and 2021-2022, respectively when compared to the I_30_N_0-100_ treatment. The highest values of TN, TP, and TK content were obtained with the I_45_N_50_-_50_, I_45_SRF_100_, and I_45_N_50_-_50_ treatments, respectively during both winter wheat-growing seasons. The lowest values of TN, TP, and TK content were all obtained with the I_30_N_0_-_100_ treatment during both winter wheat growing seasons. The interactive effect of different irrigation scheduling and N application modes significantly affected TN content, while insignificantly affected TP and TK content during both winter wheat-growing seasons (**Table 7**). The I_45_N_50-50_ and I_45_SRF_100_ are the most favorable treatments for nutrient accumulations during both winter wheat growing seasons.

**Table 7:** Interactive effect of different irrigation scheduling and nitrogen application modes on winter wheat nutrient content during 2020-2021 and 2021-2022 growing seasons.

Season	2020-2021			2021-2022		
Treatment	Total N (mg g^−1^)	Total P (mg g^−1^)	Total K (mg g^−1^)	Total N (mg g^−1^)	Total P (mg g^−1^)	Total K (mg g^−1^)
I45N0-100	27.46^gh^	3.35^de^	24.93^cd^	30.17^f^	3.83^cde^	26.99^bc^
145N25-75	36.92^bc^	3.94^bc^	28.89^abc^	38.76^bc^	4.69^b^	30.70^ab^
I45N50-50	45.85^a^	4.01^b^	32.46^a^	46.49^a^	4.56^bc^	34.00^a^
I45N75-25	32.92^cde^	3.72^bcd^	27.62^bc^	35.27^cde^	4.38^bcd^	28.55^b^
I45N100-0	29.32^efgh^	3.60^bcde^	27.35^bc^	31.41^ef^	3.71^de^	29.45^ab^
I45SRF100	40.56^b^	5.13^a^	29.90^ab^	42.55^ab^	5.62^a^	31.16^ab^
I30N0-100	26.37^h^	3.16^e^	22.25^d^	29.84^f^	3.59^e^	22.91^c^
I30N25-75	30.75^efg^	3.48^cde^	26.28^bcd^	32.66^def^	3.95^bcde^	27.61^bc^
I30N50-50	35.03^cd^	3.90^bc^	28.62^abc^	37.28^cd^	4.35^bcd^	29.80^ab^
I30N75-25	29.24^efgh^	3.47^cde^	26.04^bcd^	32.21^ef^	3.94^cde^	28.40^b^
I30N100-0	28.15^fgh^	3.45^cde^	25.34^cd^	29.98^f^	4.04^bcde^	26.46^bc^
I30-SRF100	31.70^def^	4.91^a^	26.96^bc^	33.86^def^	5.69^a^	28.13^b^
I	***	*	**	***	ns	**
N	***	***	**	***	***	**
I × N	***	ns	ns	*	ns	ns

I, Irrigation level; N, Nitrogen application mode; Each value indicates the mean of three replicates and the different letters within a column represent a significant difference at P< 0.05. Significance level = ns (non-significant at P< 0.05), * (P< 0.05), ** (P< 0.01), and *** (P< 0.001).

### Polynomial relationship between grain yield and various parameters

**Figure 7:**
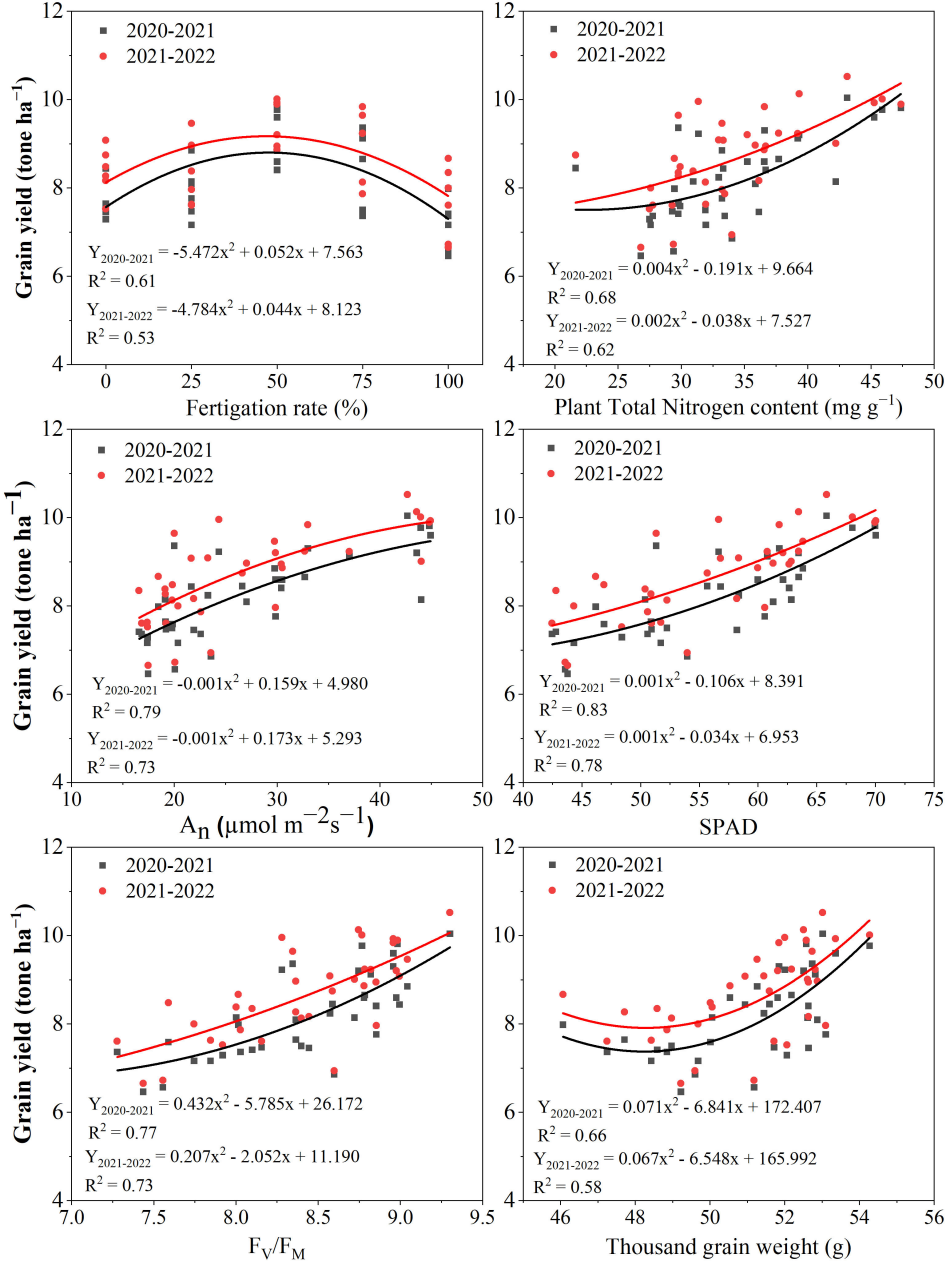
Winter wheat grain yield response to various parameters during the 2020-2021 and 2021-2022 growing seasons.

The data of winter wheat grain yield response to various parameters under different irrigation scheduling and N application modes during the 2020-2021 and 2021-2022 growing seasons are presented in **Figure 7**. It is observed that during both winter wheat-growing seasons, grain yield increases with the increase of top dressing N fertigation rate from 0 to 50% and then decreases from 50 to 100% (**Figure 7**). In this study, there are close correlations between grain yield and fertigation rate (R^2^ of 0.61 and 0.53 for both seasons), grain yield and plant TN content (R^2^ of 0.68 and 0.62 for both seasons), grain yield and A_n_ (R^2^ of 0.79 and 0.73 for both seasons), grain yield and SPAD chlorophyll content (R^2^ of 0.83 and 0.78 for both seasons), grain yield and F_V_/F_M_ (R^2^ of 0.77 and 0.73 for both seasons), and grain yield and TGW (R^2^ of 0.66 and 0.58 for both seasons). During both winter wheat-growing seasons, grain yield significantly correlated with the fertigation rate, plant TN content, A_n_, SPAD chlorophyll content, F_V_/F_M_, and TGW (**Figure 7**).

## Discussion

### Effect of different N and irrigation scheduling on crop growth and biomass accumulation

Plant growth parameters such as plant height and LAI are key characteristics of crop growth and development. N is among the most necessary plant nutrients that influence crop growth and development (Li et al., 2019a). Previous studies demonstrated that N availability positively affected crop growth and development, while water deficit negatively affected crop growth and development (Abrar et al., 2020; Si et al., 2020; Zain et al., 2021). The experimental results of the current study also indicated a significant improvement in plant height and LAI by increasing the irrigation quota. Enhancing the quota of irrigation suggests more water application, better moisture conditions in the soil, a shorter period of water deficit, and more crop evapotranspiration, which all benefit plant growth (Kharrou et al., 2011). As stated by Farooq et al. (2009), a lack of water and N induces a significant reduction in cell processes, including cell elongation, duration of cell elongation, and cell division, resulting in a reduction in leaf area. Previous studies also demonstrated that reasonable N fertilizer allocation at various wheat growth stages is essential for wheat growth under a defined fertilization rate (Zain et al., 2021; Abubakar et al., 2022). In the current study, in comparison with other irrigation and N scheduling treatments, the I_45_N_50-50_ and I_45_SFR_100_ treatments had the highest plant height and LAI during all wheat growth stages. This is in line with the findings of Abubakar et al. (2022), who reported in a recent study that split application of N in a N_50-50_ ratio is beneficial for plant height and LAI improvement during all wheat growth stages. According to Ma et al. (2021), adequate N fertilizer supply guaranteed a rational soil inorganic N distribution for meeting crop growth requirements, decreased N losses at the early growth stages, and enhanced wheat biomass accumulation at later groining stages.

Aboveground biomass (ABM) is a significant indication of crop growth and development. ABM is the material basis of grain yield because it exhaustively represents the overall contribution of plant height, LAI, and plant density. In the present study, the highest ABM was obtained under the I_45_N_50-50_ and I_45_SFR_100_ treatments during both winter wheat-growing seasons, implying that under these treatments the optimal N amount was applied for better winter wheat growth. The goal of the topdressing N fertilization was to enhance post-anthesis biomass accumulation, which is a beneficial method of enhancing crop yield (Wang et al., 2016). In the current study, I_45_N_50-50_ and I_45_SFR_100_ treatments provided the required N at crucial winter wheat growth stages, resulting in higher ABM accumulation (**Table 5**). These findings are in harmony with other researchers’ experimental results (Zhang et al., 2013; Li et al., 2016; Jha et al., 2019).

### Effect of different N and irrigation scheduling on grain yield and its components

The overall goal of this study was to develop an appropriate N application scheduling to improve winter wheat grain yield under different water regimes. In the present study, with the same irrigation regime, the N_50-50_ and SRF_100_ treatments resulted in similar grain yields during both winter wheat-growing seasons (**Table 3**). The current study also revealed that irrigating the wheat crop whenever the actual evapotranspiration (ETa-P) reaches 45 mm and split application of N at N_50-50_ is the optional fertigation method to achieve a better grain yield. Under the irrigation scheduling of 45 mm, the split application of N at N_50-50_ significantly (*P<*0.05) increased winter wheat grain yield by 25.62 and 21.63% in 2020/2021 and 2021/2022, respectively as compared to the treatment which applied 100% of the N at jointing and booting (N_0-100_). Under the same irrigation regime, the SRF_100_ increased winter wheat grain yield by 20.72 and 21.15, respectively compared to N_0-100_. Based on correlation analysis, Abubakar et al. (2022) stated that the cause for the grain yield enhancement under this fertigation method is that the treatment strongly affected the yield components. Similarly, in the present study, the winter wheat grain yield was observed to strongly correlate with the thousand-grain weight. In harmony with the findings of the present study, Liang et al. (2017) and Zhang et al. (2017) reported that excessive fertilization at the later growth stages leads to prolonged growth, poor grain filling, delayed maturity, and ultimately decreased grain yield.

Adequate irrigation scheduling and split N management are beneficial to improve winter wheat yield components. In this study, split N management played an important role in enhancing yield components, including spike lent (SL), number of grains per spike (SG), thousand-grain weight (TGW), and number of grains per 10 plants (G 10plants^−1^). Zain et al. (2021) found that managing appropriately the percentage of top-dressing N and applying more N at late stages of wheat growth helps to achieve high SG and TGW. In the present study, the I_45_N_50-50_ and I_45_SRF_100_ treatments resulted in an obvious improvement in yield components in comparison with the other experimental treatments during both winter wheat-growing seasons (**Table 4**). Results of the current study demonstrated that a 100% topdressing N is not beneficial for improving winter wheat yield components. These findings are in line with those of previous studies on the effect of split N management in winter wheat fields (Zain et al., 2021; Abubakar et al., 2022). Liu et al. (2019) also reported that reducing the basal rate and increasing the N application rate at the jointing and booting stages significantly enhanced the yield components, and ultimately, the grain yield. The yield component patterns indicated that a balancing strategy for N split application is recommended. However, the results of this study showed that the SRF_100_ treatment was very competitive with N split application.

### Effect of different N and irrigation scheduling on leaf photosynthetic parameters

Several previous studies have reported that photosynthetic capacity is the main factor determining wheat grain yield after anthesis (Tian et al., 2012; Zhang et al., 2020). Fang et al. (2018) confirmed that this capacity is directly related to the application of N fertilizer and irrigation. Consistently, the results of the current study proved that under an irrigation quota of 45 mm, a N_50-50_ ratio of basal-top-dressing N and SRF_100_ at sowing could enhance the post-anthesis winter wheat leaves photosynthetic capacity, which is directly beneficial in terms of increasing grain yield. **Figure 7** revealed a strong relationship between winter wheat grain yield and photosynthetic capacity during both growing seasons. This influence may also be attributed to the fact that a reasonable basal-top dressing N ratio can improve N accumulation in leaves, which is significantly positively correlated with chlorophyll activity and improve photosynthetic capacity (Li et al., 2013).

### Effect of different N and irrigation scheduling on SPAD chlorophyll and chlorophyll fluorescence

SPAD-measured values can be used to determine leaf functional status and nutrient content (Wang et al., 2018; Yang et al., 2018; Li et al., 2019b). The current paper analyzed the distribution of winter wheat leaf *SPAD* content among treatments under different irrigation scheduling and N application modes (**Figure 5**). Kitonyo et al. (2018) reported that high *SPAD* chlorophyll values indicate a sustained high photosynthetic rate *A_n_*. Consistently, in this study, under the irrigation quota of 45 mm, the N_50-50_ ratio of basal-top-dressing simultaneously resulted in a maximum *SPAD* chlorophyll content (**Figure 5**) and maximum *A_n_* (**Figure 4**) during both growing seasons. The post-anthesis increase in the maximal photochemical efficiency of photosystem II (*F_V_/F_M_*) of wheat leaves provides further support for this hypothesis. Although previous research has shown that appropriate irrigation is an essential tool for efficient photosynthetic capacity, the current study indicates that variation in irrigation regimes had no significant influences on the *F_V_/F_M_* of wheat eaves after anthesis (**Figure 6**). However, treatment with the optimal combination N_50-50_ basal-top-dressing N ratio and irrigation scheduling at 45 mm was associated with the highest *F_V_/F_M_*. These findings suggest that the N_50-50_ basal-top-dressing N ratio and irrigation scheduling at 45 mm improve *PSII* efficiency, which could increase photosynthesis capacity by enhancing energy transport from *PSII* to *PSI*.

### Effect of different N and irrigation scheduling on nutrient content

Nitrogen (N), phosphorus (P), and potassium (K) are the three main essential nutrients that plants require for optimum growth and development. During the life cycle, an insufficiency of any of these nutrients has a deleterious impact on plant growth and development (Khalofah et al., 2022). N plays an important role in plants’ vegetative growth, synthesis of chlorophyll, and subsequently in photosynthesis (Duarah et al., 2011; Khan et al., 2012). P is involved in the released energy storage and transfer during photosynthetic activity, and its deficit delays plant maturity (Khalofah et al., 2022). In the current study, compared to the I_30_N_100-0_ treatment, the I_45_N_50-50_ treatment significantly (*P<*0.05) increased plant total N content by 38.6 and 35.51% in 2020-2021 and 2021-2022, respectively, and significantly (*P<*0.05) increased plant total K content by 21.93 and 22.18% in 2020-2021 and 2021-2022, respectively. The highest concentrations of total P were obtained under the I_45_SRF_100_ and I_30_SRF_100_ in 2020-2021 and 2021-2022, respectively (**Table 7**). Consistently with the findings of the present study, Zhang et al. (2020) found that top-dressing N application significantly increased plant N concentration under different irrigation regimes. The results of the current study are also similar to the findings of Shedeed et al. (2009), who found that split application of urea (46% N, 200 kg ha^−1^) significantly affected tomato plants’ total NPK uptake under drip irrigation scheduling. (Alhaj Hamoud et al., 2019) demonstrated that rice N, P and K uptake were affected by different irrigation regimes.

## Conclusion

To summarize, the present study investigated the effects of split N fertilizer application and different irrigation on winter wheat growth, grain yield, photosynthetic capacity, chlorophyll fluorescence, and nutrient accumulations. The two consecutive years (2020-2021 and 2021-2022) study showed that different irrigation scheduling and N application modes significantly affected winter wheat growth, yield, and photosynthetic capacity. Collectively, the results of the current study confirm that under various irrigation, splitting the urea (46% N, 240 kg ha^−1^) at 50% at sowing and 50% from jointing to booting stages positively affected drip-irrigated winter wheat. The I_45_N_50-50_ and I_45_SRF_100_ treatments resulted in the highest grain yield, aboveground biomass, net photosynthetic rate, stomatal conductance, SPAD chlorophyll content, and chlorophyll fluorescence (*F_V_/F_M_*). Therefore, this study concludes that treatments based on I_45_N_50-50_ is an optional choice for winter wheat production in the North China Plain. The present study shows that an optimized split N fertilizer application could help to sustain a better winter wheat physiological growth and yield formation. This study’s findings also indicated that using slow-release fertilizer (SRF) to replace N application is a promising method because it could offset the costs due to its single-time application of urea. Additional studies should be conducted to assess soil water-nitrogen use efficiency, soil microbial community activities, soil water and N dynamics, as well as the quantification of N losses through emissions and leaching.

## Data availability statement

The raw data supporting the conclusions of this article will be made available by the authors, without undue reservation.

## Author contributions

AH: writing—original draft preparation and investigation. AH: methodology, investigation, formal analysis, software, validation, visualization, data curation. YG and AD: methodology, conceptualization and design. SA: methodology, investigation, writing—review and editing. ZS, RK, SA, YG and AD: writing—review and editing. All authors contributed to the article and approved the submitted version.

## References

[B1] Abrar M. M. Saqib M. Abbas G. Atiq-Ur-Rahman M. Mustafa A. Shah S. A. A.. (2020). Evaluating the contribution of growth, physiological, and ionic components towards salinity and drought stress tolerance in jatropha curcas. Plants 9, 1574. doi: 10.3390/plants9111574 33203052 PMC7696781

[B2] Abubakar S. A. Hamani A. K. M. Wang G.-S. Hao L. Mehmood F. Abdullahi A. S.. (2022). Growth and nitrogen productivity of drip-irrigated winter wheat under different nitrogen fertigation strategies in the north China plain. J. Integr. Agric. doi: 10.1016/j.jia.2022.08.107

[B3] Adeel M. Farooq T. White J. C. Hao Y. He Z. Rui Y. (2021). Carbon-based nanomaterials suppress tobacco mosaic virus (TMV) infection and induce resistance in nicotiana benthamiana. J. Hazardous Materials 404, 124167. doi: 10.1016/j.jhazmat.2020.124167 33049632

[B4] Alhaj Hamoud Y. Shaghaleh H. Sheteiwy M. Guo X. Elshaikh N. A. Ullah Khan N.. (2019). Impact of alternative wetting and soil drying and soil clay content on the morphological and physiological traits of rice roots and their relationships to yield and nutrient use-efficiency. Agric. Water Manage. 223, 105706. doi: 10.1016/j.agwat.2019.105706

[B5] Allen R. G. Pereira L. S. Raes D. Smith M. (1998). Crop evapotranspiration-guidelines for computing crop water requirements-FAO irrigation and drainage paper 56 Vol. 300 (Rome: Fao), D05109.

[B6] Al-Rawajfeh A. E. Alrbaihat M. R. Alshamaileh E. M. (2021). “Characteristics and types of slow-and controlled-release fertilizers,” in Controlled release fertilizers for sustainable agriculture (Elsevier), 57–78.

[B7] Ashraf M. N. Aziz T. Maqsood M. A. Bilal H. M. Raza S. Zia M.. (2019). Evaluating organic materials coating on urea as potential nitrification inhibitors for enhanced nitrogen recovery and growth of maize (Zea mays). Int. J. Agric. Biol. 22, 1102–1108.

[B8] Blandino M. Vaccino P. Reyneri A. (2015). Late-season nitrogen increases improver common and durum wheat quality. Agron. J. 107, 680–690. doi: 10.2134/agronj14.0405

[B9] Bozkurt Y. Yazar A. Gençel B. Sezen M. S. (2006). Optimum lateral spacing for drip-irrigated corn in the Mediterranean region of Turkey. Agric. Water Manage. 85, 113–120. doi: 10.1016/j.agwat.2006.03.019

[B10] Bremner J. M. (1996). “Nitrogen-total,” in Methods of soil analysis: Part 3 chemical methods, vol. 5. , 1085–1121.

[B11] Chen J. Lü S. Zhang Z. Zhao X. Li X. Ning P.. (2018a). Environmentally friendly fertilizers: A review of materials used and their effects on the environment. Sci. Total Environ. 613, 829–839. doi: 10.1016/j.scitotenv.2017.09.186 28942316

[B12] Chen X. Wang J. Wang Z. Li W. Wang C. Yan S.. (2018b). Optimized nitrogen fertilizer application mode increased culms lignin accumulation and lodging resistance in culms of winter wheat. Field Crops Res. 228, 31–38. doi: 10.1016/j.fcr.2018.08.019

[B13] Duan J. Shao Y. He L. Li X. Hou G. Li S.. (2019). Optimizing nitrogen management to achieve high yield, high nitrogen efficiency and low nitrogen emission in winter wheat. Sci. Total Environ. 697, 134088. doi: 10.1016/j.scitotenv.2019.134088 31487591

[B14] Duarah I. Deka M. Saikia N. Deka Boruah H. (2011). Phosphate solubilizers enhance NPK fertilizer use efficiency in rice and legume cultivation. 3 Biotech. 1, 227–238. doi: 10.1007/s13205-011-0028-2 PMC3339586 22558541

[B15] El-Sayed O. M. El-Hagarey M. E. (2014). Evaluation of ultra-low drip irrigation and relationship between moisture and salts in soil and peach (Pruns perssica) yield. J. Am. Sci. 10.

[B16] Fang X. Li Y. Nie J. Wang C. Huang K. Zhang Y.. (2018). Effects of nitrogen fertilizer and planting density on the leaf photosynthetic characteristics, agronomic traits and grain yield in common buckwheat (Fagopyrum esculentum m.). Field Crops Res. 219, 160–168. doi: 10.1016/j.fcr.2018.02.001

[B17] Farooq M. Wahid A. Kobayashi N. Fujita D. Basra S. (2009). “Plant drought stress: effects, mechanisms and management,” in Sustainable agriculture (Springer), 153–188.

[B18] Gao Y. Duan A. Sun J. Li F. Liu Z. Liu H.. (2009). Crop coefficient and water-use efficiency of winter wheat/spring maize strip intercropping. Field Crops Res. 111, 65–73. doi: 10.1016/j.fcr.2008.10.007

[B19] Gui Y.-W. Sheteiwy M. S. Zhu S.-G. Batool A. Xiong Y.-C. (2021). Differentiate effects of non-hydraulic and hydraulic root signaling on yield and water use efficiency in diploid and tetraploid wheat under drought stress. Environ. Exp. Bot. 181, 104287. doi: 10.1016/j.envexpbot.2020.104287

[B20] Jackson M. (1973). Soil chemical analysis, pentice hall of India pvt. ltd., new Delhi, India 498, 151–154.

[B21] Jha S. K. Gao Y. Liu H. Huang Z. Wang G. Liang Y.. (2017). Root development and water uptake in winter wheat under different irrigation methods and scheduling for north China. Agric. Water Manage. 182, 139–150. doi: 10.1016/j.agwat.2016.12.015

[B22] Jha S. K. Ramatshaba T. S. Wang G. Liang Y. Liu H. Gao Y.. (2019). Response of growth, yield and water use efficiency of winter wheat to different irrigation methods and scheduling in north China plain. Agric. Water Manage. 217, 292–302. doi: 10.1016/j.agwat.2019.03.011

[B23] Khalofah A. Ghramh H. A. Al-Qthanin R. N. L’taief B. (2022). The impact of NPK fertilizer on growth and nutrient accumulation in juniper (Juniperus procera) trees grown on fire-damaged and intact soils. PloS One 17, e0262685. doi: 10.1371/journal.pone.0262685 35085316 PMC8794100

[B24] Khan M. Rafiq R. Hussain M. Farooq M. Jabran K. (2012). Ridge sowing improves root system, phosphorus uptake, growth and yield of maize (Zea mays l.) hybrids. Measurements 22, 309–317.

[B25] Kharrou M. H. Er-Raki S. Chehbouni A. Duchemin B. Simonneaux V. Le Page M.. (2011). Water use efficiency and yield of winter wheat under different irrigation regimes in a semi-arid region. Agric. Sci. China 2, 273–282. doi: 10.4236/as.2011.23036

[B26] Kirda C. Topcu S. Cetin M. Dasgan H. Kaman H. Topaloglu F.. (2007). Prospects of partial root zone irrigation for increasing irrigation water use efficiency of major crops in the Mediterranean region. Ann. Appl. Biol. 150, 281–291. doi: 10.1111/j.1744-7348.2007.00141.x

[B27] Kitonyo O. M. Sadras V. O. Zhou Y. Denton M. D. (2018). Nitrogen supply and sink demand modulate the patterns of leaf senescence in maize. Field Crops Res. 225, 92–103. doi: 10.1016/j.fcr.2018.05.015

[B28] Lamm F. R. Trooien T. P. (2003). Subsurface drip irrigation for corn production: a review of 10 years of research in Kansas. Irrigation Sci. 22, 195–200. doi: 10.1007/s00271-003-0085-3

[B29] Liang W. Zhang Z. Wen X. Liao Y. Liu Y. (2017). Effect of non-structural carbohydrate accumulation in the stem pre-anthesis on grain filling of wheat inferior grain. Field Crops Res. 211, 66–76. doi: 10.1016/j.fcr.2017.06.016

[B30] Li Q. Chen Y. Liu M. Zhou X. Yu S. Dong B. (2008). Effects of irrigation and planting patterns on radiation use efficiency and yield of winter wheat in north China. Agric. Water Manage. 95, 469–476. doi: 10.1016/j.agwat.2007.11.010

[B31] Li Y. Liu H. Huang G. (2016). The effect of nitrogen rates on yields and nitrogen use efficiencies during four years of wheat–maize rotation cropping seasons. Agron. J. 108, 2076–2088. doi: 10.2134/agronj2015.0610

[B32] Li Y. Song H. Zhou L. Xu Z. Zhou G. (2019b). Vertical distributions of chlorophyll and nitrogen and their associations with photosynthesis under drought and rewatering regimes in a maize field. Agric. For. Meteorol 272, 40–54. doi: 10.1016/j.agrformet.2019.03.026

[B33] Li D. Tian M. Cai J. Jiang D. Cao W. Dai T. (2013). Effects of low nitrogen supply on relationships between photosynthesis and nitrogen status at different leaf position in wheat seedlings. Plant Growth Regul. 70, 257–263. doi: 10.1007/s10725-013-9797-4

[B34] Liu Z. Gao F. Liu Y. Yang J. Zhen X. Li X.. (2019). Timing and splitting of nitrogen fertilizer supply to increase crop yield and efficiency of nitrogen utilization in a wheat–peanut relay intercropping system in China. Crop J. 7, 101–112. doi: 10.1016/j.cj.2018.08.006

[B35] Li J. Wang Y. Zhang M. Liu Y. Xu X. Lin G.. (2019a). Optimized micro-sprinkling irrigation scheduling improves grain yield by increasing the uptake and utilization of water and nitrogen during grain filling in winter wheat. Agric. Water Manage. 211, 59–69. doi: 10.1016/j.agwat.2018.09.047

[B36] Ma Q. Wang M. Zheng G. Yao Y. Tao R. Zhu M.. (2021). Twice-split application of controlled-release nitrogen fertilizer met the nitrogen demand of winter wheat. Field Crops Res. 267, 108163. doi: 10.1016/j.fcr.2021.108163

[B37] Mehrabi F. Sepaskhah A. R. (2022). Leaf nitrogen, based on SPAD chlorophyll reading can determine agronomic parameters of winter wheat. Int. J. Plant Production 16, 77–91. doi: 10.1007/s42106-021-00172-2

[B38] Misra R. (1968). Ecology workbook (Scientific publishers).

[B39] Olszewski J. Makowska M. Pszczółkowska A. Okorski A. Bieniaszewski T. (2014). The effect of nitrogen fertilization on flag leaf and ear photosynthesis and grain yield of spring wheat. Plant Soil Environ. 60, 531–536. doi: 10.17221/880/2013-PSE

[B40] Pan J. Zhao J. Liu Y. Huang N. Tian K. Shah F.. (2019). Optimized nitrogen management enhances lodging resistance of rice and its morpho-anatomical, mechanical, and molecular mechanisms. Sci. Rep. 9, 1–13. doi: 10.1038/s41598-019-56620-7 31889083 PMC6937289

[B41] Qu C.-H. Li X.-X. Ju H. Liu Q. (2019). The impacts of climate change on wheat yield in the Huang-Huai-Hai plain of China using DSSAT-CERES-Wheat model under different climate scenarios. J. Integr. Agric. 18, 1379–1391. doi: 10.1016/S2095-3119(19)62585-2

[B42] Shan P. Li D. Cai P. Zheng K. Liu H. Lu Y.. (2022). Direct fabrication of cross-linking polymer coatings from monomers *via* triethylamine gas-mediated thiol-ene chemistry for sustained fertilizers release. Prog. Organic Coatings 162, 106594. doi: 10.1016/j.porgcoat.2021.106594

[B43] Shedeed S. I. Zaghloul S. M. Yassen A. (2009). Effect of method and rate of fertilizer application under drip irrigation on yield and nutrient uptake by tomato. Ozean J. Appl. Sci. 2, 139–147.

[B44] Shen X. Wang G. Tilahun Zeleke K. Si Z. Chen J. Gao Y. (2020). Crop water production functions for winter wheat with drip fertigation in the north China plain. Agronomy 10, 876. doi: 10.3390/agronomy10060876

[B45] Si Z. Zain M. Mehmood F. Wang G. Gao Y. Duan A. (2020). Effects of nitrogen application rate and irrigation regime on growth, yield, and water-nitrogen use efficiency of drip-irrigated winter wheat in the north China plain. Agric. Water Manage. 231, 106002. doi: 10.1016/j.agwat.2020.106002

[B46] Sun W. Hamani A. K. M. Si Z. Abubakar S. A. Liang Y. Liu K.. (2022). Effects of timing in irrigation and fertilization on soil NO3–n distribution, grain yield and water–nitrogen use efficiency of drip-fertigated winter wheat in the north China plain. Water 14, 1780. doi: 10.3390/w14111780

[B47] Tayel M. Mansour H. (2013). Effect of drip irrigation circuits design and lateral lines length on: V-water and fertilizer use efficiency. Curr. Adv. Environ. Sci. 1, 1–6.

[B48] Tian Y. Chen J. Chen C. Deng A. Song Z. Zheng C.. (2012). Warming impacts on winter wheat phenophase and grain yield under field conditions in Yangtze delta plain, China. Field Crops Res. 134, 193–199. doi: 10.1016/j.fcr.2012.05.013

[B49] Tian Z.-W. Liu X.-X. Gu S.-L. Yu J.-H. Zhang L. Zhang W.-W.. (2018). Postponed and reduced basal nitrogen application improves nitrogen use efficiency and plant growth of winter wheat. J. Integr. Agric. 17, 2648–2661. doi: 10.1016/S2095-3119(18)62086-6

[B50] Wang L. Sun J. Wang C. Shangguan Z. (2018). Leaf photosynthetic function duration during yield formation of large-spike wheat in rainfed cropping systems. PeerJ 6, e5532. doi: 10.7717/peerj.5532 30280014 PMC6166621

[B51] Wang B. Zhang Y. Hao B. Xu X. Zhao Z. Wang Z.. (2016). Grain yield and water use efficiency in extremely-late sown winter wheat cultivars under two irrigation regimes in the north China plain. PloS One 11, e0153695. doi: 10.1371/journal.pone.0153695 27100187 PMC4839561

[B52] Xu J. Shi Y. Yu Z. Zhao J. (2017). Irrigation methods affect wheat flag leaf senescence and chlorophyll fluorescence in the north China plain. Int. J. Plant Production 11.

[B53] Yang H. Fang C. Li Y. Wu Y. Fransson P. Rillig M. C.. (2022). Temporal complementarity between roots and mycorrhizal fungi drives wheat nitrogen use efficiency. New Phytol. 236, 1168–1181. doi: 10.1111/nph.18419 35927946

[B54] Yang H. Yang J. Li F. Liu N. (2018). Replacing the nitrogen nutrition index by SPAD values and analysis of effect factors for estimating rice nitrogen status. Agron. J. 110, 545–554. doi: 10.2134/agronj2017.09.0532

[B55] Yan S. Wu Y. Fan J. Zhang F. Qiang S. Zheng J.. (2019). Effects of water and fertilizer management on grain filling characteristics, grain weight and productivity of drip-fertigated winter wheat. Agric. Water Manage. 213, 983–995. doi: 10.1016/j.agwat.2018.12.019

[B56] Ye T. Ma J. Zhang P. Shan S. Liu L. Tang L.. (2022). Interaction effects of irrigation and nitrogen on the coordination between crop water productivity and nitrogen use efficiency in wheat production on the north China plain. Agric. Water Manage. 271, 107787. doi: 10.1016/j.agwat.2022.107787

[B57] Zain M. Si Z. Li S. Gao Y. Mehmood F. Rahman S.-U.. (2021). The coupled effects of irrigation scheduling and nitrogen fertilization mode on growth, yield and water use efficiency in drip-irrigated winter wheat. Sustainability 13, 2742. doi: 10.3390/su13052742

[B58] Zhang J.-H. Jian-Li L. Zhang J.-B. Cheng Y.-N. Wei-Peng W. (2013). Nitrate-nitrogen dynamics and nitrogen budgets in rice-wheat rotations in taihu lake region, China. Pedosphere 23, 59–69. doi: 10.1016/S1002-0160(12)60080-0

[B59] Zhang Y. Wang J. Gong S. Xu D. Sui J. (2017). Nitrogen fertigation effect on photosynthesis, grain yield and water use efficiency of winter wheat. Agric. Water Manage. 179, 277–287. doi: 10.1016/j.agwat.2016.08.007

[B60] Zhang Z. Zhang Y. Shi Y. Yu Z. (2020). Optimized split nitrogen fertilizer increase photosynthesis, grain yield, nitrogen use efficiency and water use efficiency under water-saving irrigation. Sci. Rep. 10, 1–14. doi: 10.1038/s41598-020-75388-9 33219232 PMC7680147

[B61] Zhang G. Zhao D. Liu S. Liao Y. Han J. (2022). Can controlled-release urea replace the split application of normal urea in China? a meta-analysis based on crop grain yield and nitrogen use efficiency. Field Crops Res. 275, 108343. doi: 10.1016/j.fcr.2021.108343

[B62] Zhang H. Zhao Q. Wang Z. Wang L. Li X. Fan Z.. (2021). Effects of nitrogen fertilizer on photosynthetic characteristics, biomass, and yield of wheat under different shading conditions. Agronomy 11, 1989. doi: 10.3390/agronomy11101989

[B63] Zhao F. Zou G. Shan Y. Ding Z. Dai M. He Z. (2019). Coconut shell derived biochar to enhance water spinach (Ipomoea aquatica forsk) growth and decrease nitrogen loss under tropical conditions. Sci. Rep. 9, 1–8. doi: 10.1038/s41598-019-56663-w 31889091 PMC6937338

[B64] Zheng X. Yu Z. Zhang Y. Shi Y. (2021). Effect of nitrogen rates on wheat photosynthesis, anatomical parameters and photoassimilate partitioning in north China plain. Int. J. Plant Production 15, 161–172. doi: 10.1007/s42106-020-00123-3

[B65] Živčák M. Olšovská K. Slamka P. Galambošová J. Rataj V. Shao H.. (2015). Application of chlorophyll fluorescence performance indices to assess the wheat photosynthetic functions influenced by nitrogen deficiency. Plant Soil Environ. 60, 210–215.

